# Extracellular Vesicles Derived from Induced Pluripotent Stem Cells Promote Renoprotection in Acute Kidney Injury Model

**DOI:** 10.3390/cells9020453

**Published:** 2020-02-17

**Authors:** Federica Collino, Jarlene A. Lopes, Marta Tapparo, Giovane G. Tortelote, Taís H. Kasai-Brunswick, Gustavo M.C. Lopes, Douglas B. Almeida, Renata Skovronova, Camila H. C. Wendt, Kildare R. de Miranda, Benedetta Bussolati, Adalberto Vieyra, Rafael Soares Lindoso

**Affiliations:** 1 Institute of Biophysics Carlos Chagas Filho, Federal University of Rio de Janeiro, 21941-902 Rio de Janeiro, Brazil; federica.collino@unipd.it (F.C.); jarlenelopes@biof.ufrj.br (J.A.L.); giovanegt@gmail.com (G.G.T.); tais@cenabio.ufrj.br (T.H.K.-B.); gugaclopes@hotmail.com (G.M.C.L.); douglas-7451@hotmail.com (D.B.A.);; 2 National Institute of Science and Technology for Regenerative Medicine-REGENERA, Federal University of Rio de Janeiro, 21941-902 Rio de Janeiro, Brazil; 3 Department of Biomedical Sciences, University of Padova, 35131 Padua, Italy; 4 National Center for Structural Biology and Bioimaging/CENABIO, Federal University of Rio de Janeiro, 21941-902 Rio de Janeiro, Brazil; 5 Department of Medical Sciences, Molecular Biotechnology Center, University of Torino, 10126 Torino, Italy; marta.tapparo@gmail.com; 6 Department of Pediatrics’ Section of Pediatric Nephrology, Tulane University School of Medicine, New Orleans, LA 70112, USA; 7 Department of Molecular Biotechnology and Health Sciences, University of Torino, 10126 Turin, Italybenedetta.bussolati@unito.it (B.B.); 8 National Institute of Science and Technology of Structural Biology and Bioimaging-INBEB, Federal University of Rio de Janeiro, 21941-902 Rio de Janeiro, Brazil; 9 Graduate Program of Translational Biomedicine/BIOTRANS, Grande Rio University, 25071-202 Duque de Caxias, Brazil

**Keywords:** iPSC, extracellular vesicles, AKI, mitochondria, ROS, kidney

## Abstract

Induced pluripotent stem cells (iPSC) have been the focus of several studies due to their wide range of application, including in cellular therapy. The use of iPSC in regenerative medicine is limited by their tumorigenic potential. Extracellular vesicles (EV) derived from stem cells have been shown to support renal recovery after injury. However, no investigation has explored the potential of iPSC-EV in the treatment of kidney diseases. To evaluate this potential, we submitted renal tubule cells to hypoxia-reoxygenation injury, and we analyzed cell death rate and changes in functional mitochondria mass. An in vivo model of ischemia-reperfusion injury was used to evaluate morphological and functional alterations. Gene array profile was applied to investigate the mechanism involved in iPSC-EV effects. In addition, EV derived from adipose mesenchymal cells (ASC-EV) were also used to compare the potential of iPSC-EV in support of tissue recovery. The results showed that iPSC-EV were capable of reducing cell death and inflammatory response with similar efficacy than ASC-EV. Moreover, iPSC-EV protected functional mitochondria and regulated several genes associated with oxidative stress. Taken together, these results show that iPSC can be an alternative source of EV in the treatment of different aspects of kidney disease.

## 1. Introduction

Stem cells have been the focus of investigation as a therapeutic tool in the treatment of several diseases, including kidney disorders [1]. Most of the studies investigated the properties of adult stem cells due to the possibility of isolating these cells from several adult tissues, due to ethical matters, and due to the low or absent tumorigenic risk associated with pluripotent stem cells [2]. Results based on the in vivo and in vitro models mimicking acute kidney injury (AKI) have shown that the presence of bone marrow and tissue-derived mesenchymal stromal cells (MSC) can support renal recovery, as well as the fact that the main mechanism by which these cells act is through the paracrine secretion [3,4]. One important component of paracrine secretion is the extracellular vesicles (EV), a heterogeneous population of small membrane vesicles, consisting of a lipid bilayer that allows cell-to-cell communication by direct transfer of nucleic acids, proteins, and lipids that can alter the target cell phenotype [5]. EV have been associated with the regulation of different physiological and pathological processes, including kidney disease. MSC-derived EV (MSC-EV) actions were initially reported in glycerol-induced AKI models [6]. The beneficial effects of bone marrow MSC-EV were similar to those observed with the administration of MSC itself, promoting proliferation and conferring resistance to apoptosis of renal tubular epithelial cells [6,7]. Moreover, MSC-EV were also shown to have an important role in the treatment of chronic kidney disease (CKD) by reducing inflammation and tissue damage, maintenance of renal function, and prevention of fibrosis [8,9].

In this context, the EV open a new perspective in the use of the induced pluripotent stem cells (iPSCs) and its use in regenerative medicine. Yamanaka and colleagues demonstrated the possibility of reprogramming a somatic cell to a pluripotent cell state through the transfection of the octamer-binding transcription factor 3/4 (Oct3/4), sex determining region Y-box 2 (Sox2), Kruppel-like factor 4 (Klf4), and cellular- master regulator of cell cycle entry and proliferative metabolism (c-Myc) genes [10]. Numerous studies have used this technology to promote investigations related to cell differentiation processes or even as a platform for highly specific pharmacological tests [11]. However, little is known about the potential of iPSCs in promoting renal tissue regeneration. Lee and co-workers demonstrated that iPSC can protect the kidney through the induction of anti-inflammatory, antioxidant, and anti-apoptotic mechanisms in an ischemia-reperfusion injury (IRI) model [12]. In addition, iPSC can release EV that carry molecules with cardioprotective properties, thus overcoming—for the first time—the negative effects of classical cell therapy associated with tumorigenesis [13]. With greater potential for self-renewal and expansion in culture compared to adult and progenitor stem cells, iPSCs may represent an interesting and viable source of EV production for the use in the treatment of kidney diseases. The aim of this study was to evaluate the protective and regenerative potential of EV from iPSC (iPSC-EV) and understand the mechanisms involved in these processes to explore the use of iPSC as a tool in regenerative medicine for kidney disease treatment.

## 2. Materials and Methods

### 2.1. Cell Culture

Human renal proximal tubule epithelial cells (RPTEC) – HK-2 cells from American Type Culture Collection (ATCC; Manassas, VA, USA) were used for in vitro experiments. The cells were cultured in Keratinocyte Serum-Free Medium (K-SFM) medium with 5% fetal calf serum (FCS; Thermo Fisher Scientific, Waltham, MA, USA) (5% CO_2_ in air at 37 °C). During the injury assays, cells were maintained in low-glucose Dulbecco’s Modified Eagle Medium (DMEM) (Thermo Fisher Scientific) in the absence of FCS. Because MSC-EV properties are well-described in renal recovery, we used human adipose-derived mesenchymal stromal cells (ASC; Lonza, Basel, Switzerland) and their EV (ASC-EV) to compare with the effects promoted by iPSC-EV. ASC were cultured in Adipose-Derived Stem Cell Growth BulletKit Medium (Lonza) (5% CO_2_ in air at 37 °C).

### 2.2. iPSC Generation and Characterization

The iPSCs were generated from fibroblast obtained from skin biopsy after informed consent of healthy donors, according to the protocol previously described in [14], with mild modifications. Briefly, skin fibroblasts were reprogrammed using the integration-free CytoTune-iPS 2.0 Sendai Reprogramming Kit (Thermo Fisher Scientific, Waltham, MA, USA), that contains Sendai virus particles bearing the four Yamanaka factors [15]. About 200,000 cells per well were plated onto a gelatin-coated six-well plate for 24 h prior to viral transduction. The cells were transduced according to the manufacturer’s protocol. Five days after transduction, cells were plated onto a 10 cm tissue culture plate, previously coated with 1.5 × 10^6^ mouse embryonic fibroblasts (MEF) cells, and fed with iPS cells’ medium supplemented with 30 ng/mL freshly added basic fibroblast growth factor (bFGF) (Thermo Fisher Scientific) every day. About 21 days after plating, well-formed colonies were picked for expansion into individual iPS cell lines. Hereafter, expansion progressed on Matrigel-coated plates (BD Biosciences, San Jose, CA, USA). Cells were kept in feeder-free maintenance medium for human embryonic stem cells and iPSC (mTeSR-1) (StemCell Technologies, Köln, Germany). The experimental protocol was approved by the research ethics board of the Clementino Fraga Filho University Hospital (38583914.7.0000.5257/933.018, Rio de Janeiro, Brazil). Another iPSC line was obtained from peripheral blood, according to the protocol described in [16], and with the approval of the Research Ethics Committee of the National Institute of Cardiology (27044614.3.0000.5272, Rio de Janeiro, Brazil).

For characterization of iPSC, qRT-PCR was performed to evaluate the mRNA levels of Sox2, Oct4, c-Myc, and Nanog homeobox (Nanog) through a High Capacity cDNA Reverse Transcription Kit (Applied Biosystems) and Power SYBR Green PCR Master Mix (Applied Biosystems, Foster City, CA, USA). Negative cDNA controls (no cDNA) were cycled in parallel with each run. qRT-PCR was performed with a ViiA 7 Real-Time PCR System (Applied Biosystems, Foster City, CA, USA). The mRNA levels present in the iPSC were compared to normal human dermal fibroblasts (NHDF; Lonza). We also evaluated the expression of pluripotency markers by immunofluorescence. The cells cultivated in coverslips were initially washed twice with 2 mL of ice-cold phosphate-buffered saline (PBS) and fixed with 4% paraformaldehyde and posteriorly permeabilized with 1% Triton X-100 (Thermo Fisher Scientific). Fixed cells were incubated with blocking solution (10% normal goat serum, 0.1% Tween 20 in PBS) before incubation with primary antibody. The antibodies used for pluripotency characterization—mouse Oct3/4 (Santa Cruz Biotechnology SC5279, Dallas, TX, USA), mouse Podocalyxin (TRA-1-60; Merck Millipore MAB4360), and mouse stage-specific embryonic antigen-4 (SSEA4; Merck Millipore MAB4304)—were incubated for 1 h at room temperature (dilution 1:100). The secondary antibodies (goat anti-mouse immunoglobulin G (IgG) Alexa 488-conjugated, or goat anti-mouse Alexa 594-conjugated, both from Thermo Fisher Scientific) were incubated with the cells for 2 h at room temperature (dilution 1:200). The nuclei were stained with 4′,6-diamidino-2-phenylindole (DAPI; Santa Cruz Biotechnology, Santa Cruz, CA, USA). Coverslips were mounted on glass slides with Vectashield mounting media (Vector Laboratories, Burlingame, CA, USA) and sealed. Cells were visualized by using a Zeiss confocal LSM710 microscope (Oberkochen, Germany) provided with the appropriate laser beams.

### 2.3. EV Isolation and Characterization

The supernatant from iPSC, cultured for 24 h in mTeSR-1 medium, and ASC, cultured for 24 h in Roswell Park Memorial Institute (RPMI) medium (Thermo Fisher Scientific, Waltham, MA, USA) cultures was collected and centrifuged at 300× *g*, followed by centrifugation of 2000× *g* for 20 min to remove cells and debris. The supernatants were ultracentrifuged at 100,000× *g* (Optima L-90K ultracentrifuge; Beckman Coulter, Brea, CA, USA) for 2 h at 4 °C, and the pellets were then resuspended in RPMI and submitted to the second ultracentrifugation at 100,000× *g*, for 2 h at 4 °C. The final pellet containing ASC-EV or iPSC-EV were resuspended in RPMI and stored at -80 °C in aliquots. The characterization of EV was performed in accordance with the MISEV 2018 guidelines [17]. The size and number of isolated EV were analyzed by a ZetaView-Nanoparticle Tracking Video Microscope PMX-120 instrument (Particle Metrix, Germany).

Transmission electron microscopy (TEM) was performed to characterize the iPSC-EV. Isolated iPSC-EV resuspended in PBS adhered onto glow-discharged formvar-coated copper grids 300 mesh (EMS, Hatfield, PA, USA) for 10 min. After removal of excess solution using Whatman no. 1 filter paper (Thermo Scientific), grids were negatively stained with 1% aurothioglucose (USP) in water for 30 s, dried, and examined in a Tecnai-Spirit TEM (Thermo Scientific).

An initial characterization of iPSC-EV markers was performed by using an ExoView Tetraspanin Kit (NanoView Bioscience, Boston, MA, USA). Each chip was coated with CD9, CD81, CD63, CD105, CD44 antibodies and MIgG, MIgG2a, MIgG2b control antibodies. The chips were incubated with different EV samples overnight, and protected from the light by using 35μL of EV suspension diluted at 1:100. After multiple washing steps, the chips were analyzed in ExoView R100 imaging platform (NanoView Bioscience, Boston, MA, USA). Further characterization of iPSC-EV markers was performed using the bead-based multiplex exosome flow cytometry assay (MACSPlex Exosome Kit human, Miltenyi Biotec, Bergisch Gladbach, Germany). Shortly after, samples were diluted with the MACSPlex buffer to a final concentration of 4–20 µg of protein and a final volume of 120 µL. A total of 15 µL of the MACSPlex Exosome Capture beads, which contain 39 different antibody-coated beads, and 15 µL of MACSPlex Exosome Detection Reagent cocktail, was added. After the incubation of 1 h at room temperature while being protected from the light on an orbital shaker, 1 mL of MACSPlex buffer was added and all samples were centrifuged at room temperature at 3000× *g* for 5 min. Then, the samples were washed with 1 mL of the MACSPlex, and incubated at room temperature protected from the light on an orbital shaker for 15 min followed by a wash at 3000× *g* for 5 min. A total of 150 µL of the samples were transferred to the flow cytometry tubes and characterized by using BD FACSCelesta flow cytometer (BD Biosciences, Franklin Lakes, NJ, USA).

Western blot analysis was also performed on EV previously lysed in Radioimmunoprecipitation (RIPA) buffer (Sigma-Aldrich, St. Louis, MO, USA) using the following primary antibodies: CD63 (sc-5275; 1:50; Santa Cruz Biotechnology, Dallas, TX, USA) and CD81 (sc-70803; 1:50; Santa Cruz Biotechnology). The secondary antibody anti-mouse IgG-horseradish peroxidase (HRP) (NA931, 1:10,000; GE Healthcare, Buckinghamshire, United Kingdom) was used, and proteins were detected by chemiluminescence using the electrogenerated chemiluminescence (ECL) system (GE Healthcare) and ChemiDoc XRS+ (Bio-Rad, Hercules, CA, USA).

### 2.4. In Vitro Injury Model and Cell Death Analysis

RPTEC were incubated with K-SFM medium with 5% FCS until reaching 70–80% confluence. The cells were then washed three times with PBS and cultured in low-glucose DMEM with no FCS for 24 h under hypoxic condition (37 °C, 1% O_2_, 5% CO_2_) without (HPX) or with the presence of EV (HPX+ASC-EV or HPX+iPSC-EV), 1.2 × 10^4^ vesicles per renal cell. Lower amounts of EV were tested, but no effect was observed (data not shown). After this period, the RPTEC were cultured for another 24 h under normal culture condition (37 °C, 21% O_2_, 5% CO_2_) to mimic the reperfusion phase. For control condition (CTR), the RPTEC were cultured in low-glucose DMEM with no FCS for 48 h under normal culture condition (37 °C, 21% O_2_, 5% CO_2_). The cell death analysis was performed using a Dead Cell Apoptosis Kit with annexin V fluorescein isothiocyanate (FITC) and propidium (PI) (Thermo Fisher Scientific) and fluorescent intensity was measured by flow cytometry with a BD Accuri C6 Plus instrument with CFlow Plus software (BD Biosciences, San Jose, CA, USA).

### 2.5. Measurement of Functional Mitochondrial Mass inside RPTEC

To measure the mitochondrial mass and membrane potential (ΔΨm), we used MitoTracker Mitochondrion-Selective Probes. To evaluate the alterations in mitochondrial mass inside the cells, we used MitoTracker Green FM, a fluorescent dye that stains mitochondria. In addition, to evaluate the loss of ΔΨm, we used the MitoTracker Orange CMTMRos, a fluorescent dye that accumulates only in mitochondria that present intact ΔΨm. After RPTEC were submitted to their respective experimental conditions (CTR, HPX, HPX+iPSC-EV, HPX+ASC-EV), the cells were washed three times with PBS and then harvested. The cells from each condition were divided into three groups for staining: (i) 15 min incubation with 50 nM MitoTracker Green FM dye; (ii) 15 min incubation with 50 nM MitoTracker Orange CMTMRos dye; and (iii) no dye, as a negative control. After incubation, cells were washed with PBS, and fluorescent intensity was measured by flow cytometry with a BD Accuri C6 Plus instrument and CFlow Plus software (BD Biosciences).

### 2.6. AKI In Vivo Model

The animal experiments were approved by the Ethics Committee on the Use of Animals in Scientific Experimentation of Federal University of Rio de Janeiro (Protocol 043/19). Male Wistar rats (200–250 g) were purchased from the Animal Facility of the Institute of Biomedical Sciences of the University of São Paulo, São Paulo, Brazil. Before surgical procedures, the animals were anaesthetized by intraperitoneal injection of xylazine (Bayer S.A., São Paulo, Brazil; 5 mg/kg) and ketamine (Cristália, Itapira, Brazil; 50 mg/kg) [18]. The animals were distributed into four groups: (1) the SHAM group—the rats were submitted to surgical procedures without the ischemic injury; (2) the IRI group—the rats were submitted to bilateral renal arterial clamping (45 min) followed by reperfusion with immediate subcapsular injection of PBS into each kidney after removal of the clamps; (3) the IRI+ iPSC-EV group—the rats were submitted to bilateral renal arterial clamping and received subcapsular injection on both kidneys of 1 × 10^9^ EV derived from iPSC at the beginning of reperfusion in each kidney; (4) the IRI+ASC-EV group—the rats were submitted to the same surgical procedures followed by subcapsular injection into each kidney of 1 × 10^9^ EV derived from ASC. The doses of EV were defined on the basis of previous works [8,18,19] and from other works wherein MSC-EV promoted protective effects in different kidney injury models. The subcapsular injection was chosen to observe a direct effect of EV in the kidney and to guarantee the delivery of the entire amount of EV. After 72 h, the animals were euthanized by intraperitoneal injection of a high dose of xylazine (30 mg/kg) and ketamine (200 mg/kg). The 72 h reperfusion period was shown to promote renal tissue damage, where changes in the architecture and trigger in the inflammatory response could be observed [18]. For kidney functional analysis, blood samples were collected for measurement of blood urea nitrogen (BUN) and creatinine by using the respective kits for colourimetric assays (Bioclin, Belo Horizonte, Brazil) [18]. The kidneys were collected and processed for histological and molecular analysis.

### 2.7. Histological Analysis

To evaluate tubular damage in the kidneys, 5 μm-thick paraffin kidney sections were stained with hematoxylin and eosin (Sigma-Aldrich). Images (20× objective) were taken from 15 fields of each kidney, and the tubular injury was analyzed by the number of luminal hyaline casts and number of tubules that presented denudation of basement membrane in the cortical region [20]. The injury score was given by the mean of the number of casts and denuded tubule identified in each of the 15 photomicrographs of each kidney.

### 2.8. Immunohistochemistry Analysis

Immunohistochemistry for cell proliferation and macrophage infiltration was performed using antibodies against PCNA (Agilent Technologies) and ED-1/CD68 (Bio-Rad), respectively [18]. To evaluate cell death, apoptosis was measured by Terminal deoxynucleotidyl transferase dUTP nick end labeling (TUNEL) assay (ApopTag Apoptosis Detection Kit; Millipore Inc., Billerica, MA, USA). Sections were incubated with isotype-specific immunoglobulins as negative controls. Histomorphometry analyses used a computer-assisted image analysis system (Nikon Eclipse E-800 microscope connected to a computer by a digital camera Evolution, Media Cybernetics Inc., Tallahassee, FL, USA) coupled to the software Q-Capture 2.95.0 (Silicon Graphic Inc., Milpitas, CA, USA). A total of 15 high-quality photomicrographs were captured from non-overlapping renal cortical region with a 20× or 40× objective lens.

### 2.9. RT^2^ Profiler PCR Array and qRT-PCR Confirmation

Total RNA was isolated from cortical renal specimen using the mirVana RNA isolation kit (Ambion). The RNA integrity and quality were evaluated using RNA 6000 Nano Chip and Agilent 2100 Bioanalyzer (Agilent Technologies). Only RNA integrity numbers (RINs) >8.0 in the RNA sample were considered adequate for further studies.

The rat profile analysis was conducted on different experimental groups (*n*  =  3 SHAM; *n*  =  5 IRI; *n* = 6 IRI+iPSC-EV), pooling together 2-3 animals per treatment. cDNA was then synthesized from 1 µg of RNA input and the expression of 84 oxidative stress-related genes was examined using the RT2 Profiler PCR array rat samples (Qiagen, Valencia, CA, USA) according to the manufacturer’s instructions using the Applied Biosystems 7900HT real-time PCR instrument. Data analysis was performed using Expression Suite software (Thermo Fisher Scientific) and the expression levels of the mRNA of each gene were normalized using different housekeeping genes – glyceraldehyde 3-phosphate dehydrogenase (GAPDH), Actin-like protein (ACT B), beta-2 microglobulin (B2M), lactate dehydrogenase A (LDHA), hypoxanthine phosphoribosyltransferase 1 (HPRT1), and ribosomal protein lateral stalk subunit P1 (RPLP1). Up-regulated or down-regulated transcripts with a fold change ≥ 1.5 with respect to IRI condition (Table S1) were taken into consideration only when observed in more than two arrays. Functional annotation enrichment analysis was performed using Ingenuity pathway analysis (IPA) software (Redwood City, CA USA).

To compare the regulatory capacity of iPSC-EV to the ASC-EV, we assessed the mRNA levels in RPTEC by quantitative real-time PCR using a High Capacity cDNA Reverse Transcription Kit (Thermo Fisher Scientific) and the Power SYBR Green PCR Master Mix (Thermo Fisher Scientific). Negative cDNA controls (no cDNA) were cycled in parallel with each run. Quantitative real-time polymerase chain reaction (qRT-PCR) was performed using a ViiA 7 Real-Time PCR System (Thermo Fisher Scientific). The sequence-specific oligonucleotide primers were all obtained from MWG-Biotech AG, Ebersberg, Germany (www.mwg-biotech.com) (Table S2).

### 2.10. Statistical Analysis

Statistical analyses used the Student *t*-test or the one-way analysis of variance (ANOVA) test with Tukey’s post-test. Statistical significance was set at *p* < 0.05. Data were analyzed using the GraphPad Prism 5.0 program. The data are expressed as mean ± standard error of the mean (SEM).

## 3. Results

### 3.1. Pluripotency of iPSC Were Maintained during EV Collection

**Figure 1:**
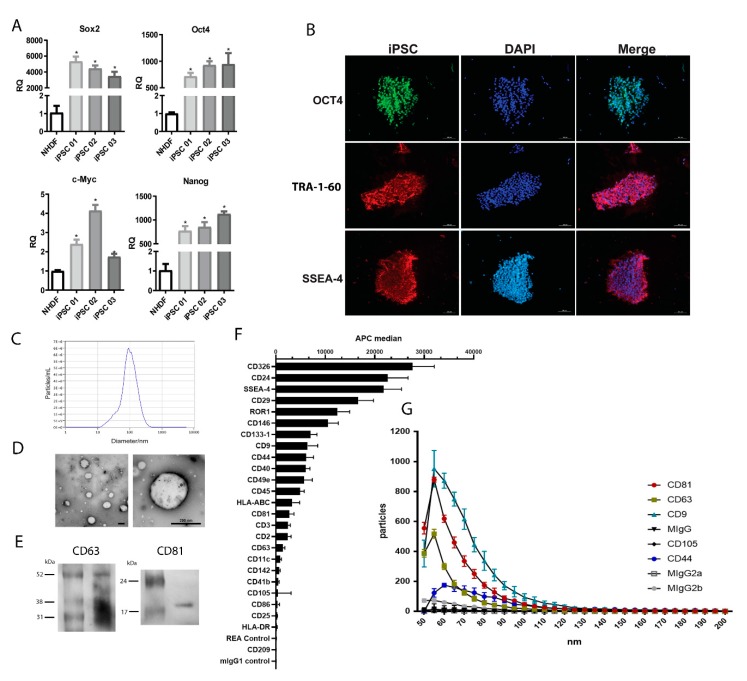
Characterization of induced pluripotent stem cells (iPSC) and iPSC-derived extracellular vesicles (iPSC-EV). (**A**) The graph indicates the mRNA level of transcription factors associated with pluripotency *SOX2, OCT4, C-MYC,* and *NANOG* in iPSC generated from three different donors (iPSC1, 2, and 3). The ordinate indicates the relative quantification of mRNA levels with respect to normal human dermal fibroblasts (NHDF). The abscissa indicates the cell types. Data represent mean ± SEM ( * indicates statistical difference to the NHDF group, *p* <0.05 assessed by one-way ANOVA followed by Tukey’s test). (**B**) Immunofluorescent staining of iPSC for pluripotency markers. The left column shows the positive staining for OCT4 (upper image) in green, TRA-1-60 (central image) in red, and SSEA-4 (lower image) in red. The central column shows the nuclei stained with DAPI in blue. The right column shows the overlap of the two previous respective images. (**C**) Representative nanoparticle tracking analysis (NTA) of (left) iPSC-EV isolated (white dots) from the culture supernatant. The right image shows the iPSC-EV size distribution and concentration, as indicated in the abscissa (diameter/nm) and in the ordinate (particles/mL). (**D**) Electron microscopy images of isolated iPSC-EV in different magnifications (scale bars = 200 nm). (**E**) Representative image of western blot of CD63 and CD81 expression in iPSC-EV. (**F**) Analysis of iPSC-EV markers with MACSPlex Exosome kit. The graph shows the median fluorescence intensity of each marker, with the background correction. The abscissa indicates the APC fluorescence intensity levels (n = 4). (**G**) Correlation between size and positivity for exosome markers of iPSC-EV. The graph shows in the ordinate the number of vesicles that are positive for each marker and the abscissa shows the diameter of vesicles. Each condition is indicated on the right according to the symbol (n = 3).

The collection of iPSC-EV was made with the supernatant of iPSC cultured for 24 h. To confirm the pluripotency state of the cell under our culturing protocol, we evaluated the pluripotency markers by qRT-PCR in comparison to NHDF. The obtained results showed that all three iPSC lines presented elevated mRNA levels of the pluripotency markers (Sox2, Oct4, c-Myc, and Nanog) that were absent in NHDF (Figure 1A). Immunofluorescent characterization was also performed to confirm the pluripotency state of iPSC (Figure 1B). The iPSC were positive for Oct4, TRA-1-60, and SSEA-4. The last two makers were originally described in embryonal carcinoma cells and are considered makers for iPSC characterization [21]. With these results, we could confirm that the EV collected were derived from iPSC.

The size distribution analysis showed a heterogeneous population of EV produced by iPSC, ranging from 32 nm to 229 nm, with a mean value of 119 nm (Figure 1C). The EV population isolated from ASC presented a size distribution that ranged from 47 nm to 211 nm, with a mean value of 125 nm (data not shown). TEM analysis revealed a classical circular morphology of iPSC-EV and confirmed the heterogeneous size of the population (Figure 1D). Western blot analysis showed the presence of exosome markers CD63 and CD81 in the iPSC-EV (Figure 1E). Moreover, a deeper analysis in the iPSC-EV markers was performed though a wide screening of surface markers by using a bead-based multiplex exosome flow cytometry assay (Figure 1F). The obtained results confirmed the presence of classical exosome markers (CD9, CD63, and CD81). Moreover, iPSC-EV were enriched of different embryonic markers – CD133-1, CD326, SSEA-4, CD24, and Receptor Tyrosine Kinase Like Orphan Receptor 1 (ROR1) – showing that the EV carry molecules associated with their cell of origin. In order to better characterize the iPSC-EV, we also performed NanoView analysis, showing the size distribution of iPSC-EV correlated to its size diameter. The results confirmed the presence of a population of exosome positive for CD9, CD63, and CD81 with size ranging between a diameter of 50-110 nm. It is worth mentioning that a small population of iPSC-EV showing positivity for the CD44 marker with the size ranging from 55 to 85 nm was detected.

### 3.2. iPSC-EV Promoted a Protective Effect against Hypoxia-Reoxygenation Injury in RPTEC

**Figure 2:**
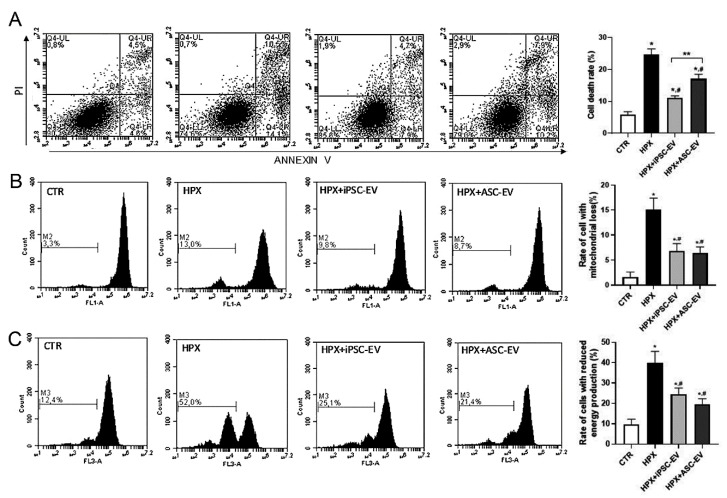
iPSC-EV reduced cell death and mitochondria damage in renal cells submitted to hypoxia-reoxygenation injury. (**A**) Representative flow cytometry analyses of renal cells stained for annexin V (ANX V)/ propidium (PI) under the different experimental conditions. From left to right panels: CTR (cells cultured in normal condition), HPX (cells submitted hypoxia-reoxygenation injury), HPX+iPSC-EV (cells submitted to injury in the presence of iPSC-EV), and HPX+ASC-EV (cells submitted to injury in the presence of ASC-EV). The graph in the right shows the quantification of flow cytometry analyses in determining the rate of cell death of renal cells in all groups (*n* = 5). (**B**) Analyses of alterations in renal cells’ mitochondria mass. The panels are representative flow cytometry analyses of renal cells incubated with the mitochondria fluorescent marker MitoTracker Green FM. The abscissa indicates the distribution of fluorescence intensity given by the respective mitochondria mass in treated cells. The ordinate indicates the number of events analyzed. M2 indicates the percentage of cells with reduced fluorescence intensity and, therefore, reduction in mitochondria mass. The graph in the right shows the percentage of the total cell population that presented a reduction of mitochondria mass, as indicated in the ordinate. The experimental group conditions are indicated in the abscissa (*n* = 5). (**C**) Analyses of alterations in renal cells’ mitochondria potential (ΔΨm). The panels are representative flow cytometry analyses of renal cells incubated with the mitochondria fluorescent marker MitoTracker Orange CMTMRos. The abscissa indicates the distribution of fluorescence intensity given by the respective mitochondria with intact ΔΨm. The ordinate indicates the number of events analyzed. M3 indicates the percentage of cells with reduced fluorescence intensity and, therefore, reduction in mitochondrial ΔΨm. The graph in the right shows the percentage of the total cell population that presented a reduction of mitochondria mass with intact ΔΨm, as indicated in the ordinate. The experimental group conditions are indicated in the abscissa (*n* = 5). Data represent mean ± SEM (* indicates statistical difference to CTR group; # indicates statistical difference to HPX group; ** indicates statistical difference to HPX+iPSC-EV group; *p* <0.05 assessed by one-way ANOVA followed by Tukey’s test).

To evaluate the therapeutic properties of iPSC-EV, we submitted RPTEC cells to hypoxia-reoxygenation injury (HPX) that mimicked the IRI (Figure 2A). HPX RPTEC presented an increase in cell death (25% ± 0.4% of dead cells) when compared to the control condition (CTR, 6% ± 0.3% of dead cells). The presence of iPSC-EV (HPX+iPSC-EV) in the culture led to considerable protection of RPTEC (11% ± 0.3% of dead cells). Such effect was stronger than the one promoted by ASC-EV (HPX+ASC-EV), which also promoted a reduction in cell death in comparison with HPX conditoin (17% ± 0.6% of dead cells).

### 3.3. Protective Effect of iPSC-EV Was Associated with Maintenance of Functional Mitochondrial Mass

One important aspect of renal tissue damage during IRI is the mitochondria alteration. Because mitochondrial damage can trigger different cell death pathways and lead to a reduction in the intracellular ATP levels [22], we wanted to observe if the reduction in cell death rate promoted by iPSC-EV could be related to the mitochondria protection (Figure 2B,C). Analysis of fluorescence intensity of RPTEC cells stained with MitoTracker Green FM and MitoTracker Orange CMTMRos (mitochondrial mass and membrane potential, respectively) revealed that RPTEC presented a reduction in mitochondrial mass after hypoxia-reoxygenation injury (HPX = 15.1% ± 1.7% of total cells) when compared to CTR cells (1.6% ± 0.6% of total cells). The impact of the injury can be better observed when we analyzed the rate of cells that presented a reduction in ΔΨm (CTR = 9.1% ± 2.2% of total cells; HPX = 37.2% ± 5.5% of total cells). Such a result indicated that hypoxia-reoxygenation injury can directly affect mitochondrial mass, also altering the mitochondrial ΔΨm inside RPTEC. Incubation of iPSC-EV partially limited the cells affected by the injury, presenting a significant reduction of the loss of mitochondrial mass and also of ΔΨm with respect to HPX condition (mitochondrial mass—HPX+iPSC-EV = 6.8% ± 1.2% of total cells; ΔΨm—HPX+iPSC-EV = 22.3% ± 2.9% of total cells). Such reduction was also observed when RPTEC were incubated with ASC-EV (mitochondrial mass—HPX+ASC-EV = 6.4% ± 1.3% of total cells; ΔΨm—HPX+ASC-EV = 20.5% ± 2.7% of total cells). EV seem to act in a way that protects mitochondria from damage by maintaining functional mitochondria that can restore cell energy supply, therefore resulting in renal cell survival.

### 3.4. iPSC-EV Promoted the Protection of Renal Tissue and Kidney Function in IRI

**Figure 3:**
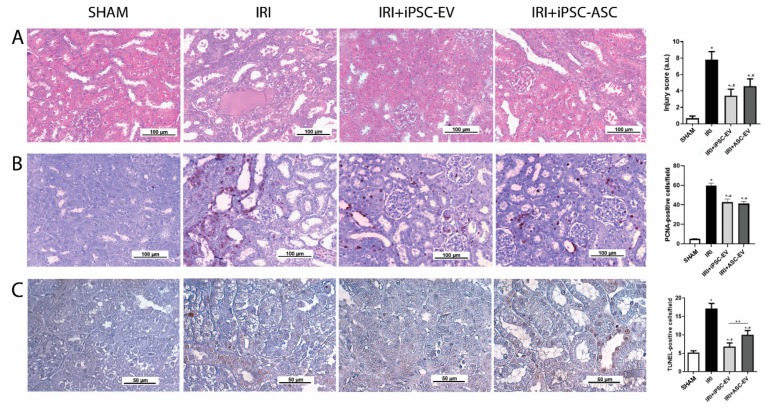
Subcapsular injection of iPSC-EV promoted the protection of renal tissue against ischemia-reperfusion injury (IRI). (**A**) Representative photomicrographs show the kidney cortical sections stained with hematoxylin/eosin (H/E) in the different experimental conditions (scale bar = 100 µm). From the left image to the right: SHAM indicates false-operated animals; IRI represents the animals submitted to ischemia-reperfusion injury by the bilateral renal arterial clamping; IRI+iPSC-EV indicates the animals submitted to renal ischemia and that received subcapsular injection of iPSC-EV into the kidney just after the clamp release; IRI+ASC-EV indicates the animals submitted to renal ischemia and that received subcapsular injection of ASC-EV into the kidney just after the clamp release. The graph shows the score of tubular lesions in each condition. SHAM, *n* (kidneys) = 4; IRI, *n* (kidneys) = 8; IRI+iPSC-EV, *n* (kidneys) = 16; IRI+ASC-EV, *n* (kidneys) = 6. (**B**) Representative photomicrographs of immunohistochemistry for PCNA (scale bar = 100 µm). The graph in the right shows the quantification of the number of PCNA-positive cells per field from immunohistochemistry. SHAM, *n* = 10 fields for 6 kidneys; IRI, *n* = 10 fields for 7 kidneys; IRI+iPSC-EV, *n* = 10 fields for 16 kidneys; IRI+ASC-EV, *n* = 10 fields for 6 kidneys. (**C**) Representative photomicrographs of immunohistochemistry for TUNEL (scale bar = 50 µm). The graph in the right shows the quantification of the number of TUNEL-positive cells per field from immunohistochemistry. SHAM, *n* = 10 fields for 6 kidneys; IRI, *n* = 10 fields for 7 kidneys; IRI+iPSC-EV, *n* = 10 fields for 16 kidneys; IRI+ASC-EV, *n* = 10 fields for 6 kidneys. Data represent mean ± SEM (* indicates statistical difference to SHAM group; # indicates statistical difference to IRI group; ** indicates statistical difference to IRI+iPSC-EV group; *p* <0.05 assessed by one-way ANOVA followed by Tukey’s test).

To assess the role of EV-iPSC in the kidney, we performed in vivo experiments using a bilateral renal arterial clamping model (Figure 3). Histological analyses of cortical kidney sections revealed that animals submitted to IRI presented damaged tubules and accumulation of hyaline casts, which were both almost absent in SHAM animals, as measured by the injury score (Figure 3A). Administration of iPSC-EV after removal of the clamping resulted in a 56% reduction of the injury score (IRI+iPSC-EV). ASC-EV-injected animals also presented a substantial reduction of 40% in renal tissue damage. Although the iPSC-EV protective effect was stronger, no significant difference was observed between the two animal groups treated with EV.

The evaluation of cell death rate was measured by the quantification of TUNEL-positive cells in cortical kidney sections (Figure 3B). The kidneys of IRI animals showed a significant increase in the number of dead tubular cells with respect to SHAM (SHAM = 5.1 ± 0.2 TUNEL-positive cells/field; IRI = 17.1 ± 0.5 TUNEL-positive cells/field). The subcapsular injection of iPSC-EV promoted, also in the in vivo model, a consistent reduction in the number of dead cells (IRI+iPSC-EV = 6.8 ± 0.2 TUNEL-positive cells/field). Such an effect was stronger than the one observed by ASC-EV administration (IRI+iPSC-EV = 9.9 ± 0.5 TUNEL-positive cells/field), although the last treatment was still effective in the reduction of tubular cell death.

A similar response was also observed when the proliferative rate in renal tubular cells was analyzed (Figure 3C). Because IRI promoted cell death and tissue damage, the proliferative response associated with the renal tissue attempt to recover resulted in the increase in the number of PCNA-positive tubular cells in the cortical region of kidney (IRI = 59.5 ± 0.9 PCNA-positive cells/field) when compared with SHAM group (SHAM = 4.9 ± 0.2 PCNA-positive cells/field). Administration of iPSC-EV and ASC-EV resulted in a significant reduction in the number of proliferating cells (IRI+iPSC-EV = 42.5 ± 0.9 TUNEL-positive cells/field; IRI+ASC-EV = 41.1 ± 0.9 TUNEL-positive cells/field). Such results indicate that the decrease of proliferative rate was associated with the tissue protection mediated by iPSC-EV.

**Figure 4:**
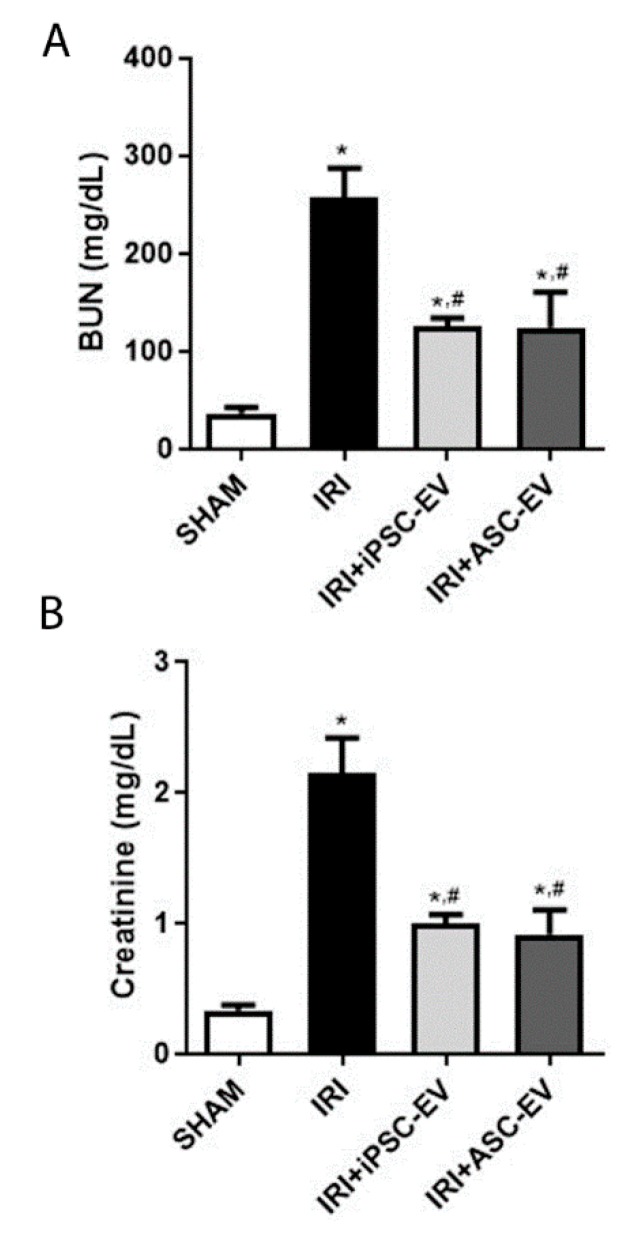
Administration of iPSC-EV after ischemia promoted the protection of kidney function. (**A**) Measurement of blood urea nitrogen (BUN) levels of the animals submitted to the different experimental conditions. The abscissa indicates each experimental groups: SHAM, IRI, IRI+iPSC-EV, and IRI+ASC-EV. The ordinate indicates the concentration of BUN (mg/dL). (**B**) Measurement of creatinine levels in the blood of the animals submitted to the different experimental conditions, as indicated in the abscissa. The ordinate indicates the creatinine concentration in the blood (mg/dL). In both analyses, SHAM, *n* = 4; IRI, *n* = 5; IRI+iPSC-EV, *n* = 11; IRI+ASC-EV, *n* = 5. Data represent mean ± SEM (* indicates statistical difference to SHAM group; # indicates statistical difference to IRI group; *p* <0.05 assessed by one-way ANOVA followed by Tukey’s test).

Analyses of renal function were assessed by the measurement of creatinine and BUN levels in the blood (Figure 4). The animals submitted to IRI presented increased and pathological levels of blood creatinine and BUN, indicating a reduction in kidney function when compared with SHAM group. Injection of iPSC-EV promoted significant conservation of kidney functions, as observed by the reduction of both parameters. The same was observed when ASC-EV were administered subcapsularly and no statistical difference was observed between iPSC-EV and ASC-EV groups.

### 3.5. iPSC-EV Promote the Reduction in Macrophage Infiltration

**Figure 5:**
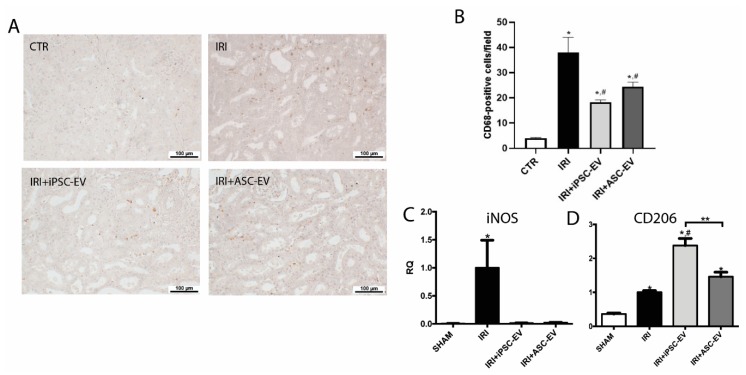
iPSC-EV promoted the reduction of the inflammatory response induced by IRI. (**A**) Representative photomicrographs of the renal tissue stained for ED-1 by immunohistochemistry. From the left image to the right: SHAM, IRI, IRI+iPSC-EV, and IRI+ASC-EV (scale bars = 100 µm). (**B**) Quantification of the number of CD68-positive cells/field. SHAM, *n* = 10 fields for 4 kidneys; IRI, *n* = 10 fields for 5 kidneys; IRI+iPSC-EV, *n* = 10 fields for 8 kidneys; IRI+ASC-EV, *n* = 10 fields for 4 kidneys. (**C**) mRNA levels of iNOS and (**D**) CD206 in renal tissue expressed as relative quantification (RQ) with respect to the IRI group (SHAM, *n* = 3; IRI, *n* = 3; IRI+iPSC-EV, *n* = 5; IRI+ASC-EV, *n* = 3). Data represent mean ± SEM (* indicates statistical difference to SHAM group; # indicates statistical difference to IRI group; ** indicates statistical difference to IRI+iPSC-EV group; *p* <0.05 assessed by one-way ANOVA followed by Tukey’s test).

Macrophages are critical elements in the inflammatory response associated with IRI, and we aimed to see if iPSC-EV could also contribute to the reduction of macrophage infiltration (Figure 5). We analyzed the number of macrophage (ED-1-positive cells) presented in the renal tissue in the different experimental conditions (Figure 5A,B). A small number of ED-1-positive cells were identified in the kidney of SHAM group (SHAM = 4 ± 0.3 ED-1-positive cells/field), whereas IRI resulted in a noteworthy increase in the number of infiltrating macrophages (IRI = 37.9 ± 6.1 ED-1-positive cells/field). As a result of iPSC-EV administration, the number of macrophages significantly decreased (IRI+iPSC-EV = 18.3 ± 0.9 ED-1-positive cells/field). ASC-EV also led to a significant reduction in the number of infiltrated macrophages (IRI+ASC-EV = 24.4 ± 1.8 ED-1-positive cells/field). In order to understand if the macrophages accumulated in the kidney had a pro-inflammatory role, we analyzed the mRNA levels of inducible nitric oxide synthase (iNOS), expressed in M1-polarized macrophages and the mRNA levels of macrophage mannose receptor (CD206), known to be upregulated in M2-polarized macrophage. The obtained results showed that mRNA levels of iNOS presented a 200-fold increase in the renal tissue submitted to IRI with respect to SHAM condition (Figure 5C). Treatment with iPSC-EV was able to completely abrogate this increase. The ASC-EV were as efficient as iPSC-EV, resulting in mRNA levels reduced almost to the levels found in SHAM. The CD206 mRNA levels were shown to be increased in the kidney after IRI (≈3-fold increase with respect to SHAM group), and the treatment with iPSC-EV after ischemic insult led to a stronger increase (≈2.4-fold in respect to IRI) (Figure 5D). In this case, CD206 was also upregulated in ASC-EV-treated rats, but they did not show statistical differences with respect to the IRI group (≈1.4- fold increase with respect to IRI). ASC-EV were also able to reduce the mRNA levels of iNOS; however, the increase of mRNA of CD206 was not significantly different with respect to IRI (Figure 5C,D, respectively). Such results indicate that macrophages were present in the renal tissue after IRI showed a pro-inflammatory phenotype.

### 3.6. iPSC Promoted Kidney Protection through Reduction of Oxidative Stress after IRI

**Figure 6:**
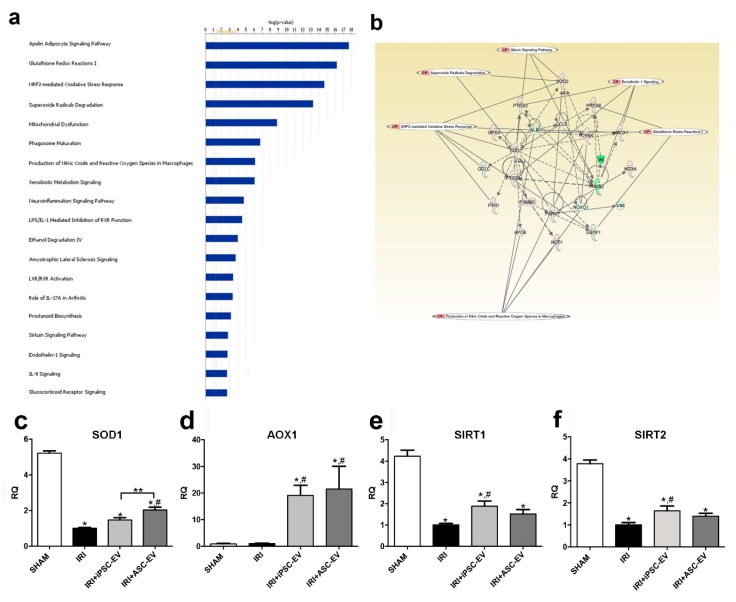
The protective effect of iPSC-EV was related to the modulation of anti-oxidative stress genes in renal tissue. The analysis performed using Ingenuity pathway analysis (IPA) showed (**a**) the enriched biological processes associated with the modulated genes by iPSC-EV administration, indicated in the horizontal bars and ordered by their significance. (**b**) IPA network analysis generated by the modulated genes by iPSC-EV in renal tissue interconnected with relevant nodes involved in the most representative biological pathways associated with the EV treatment. qRT-PCR analysis of mRNA levels of (**c**) superoxide dismutase 1 (SOD1), (**d**) aldehyde oxidase-1 (AOX1), (**e**) significantly up-regulate sirtuin 1 (SIRT1), and (**f**) SIRT2 in renal tissue expressed as relative quantification (RQ) with respect to IRI (SHAM, *n* = 3; IRI, *n* = 3; IRI+iPSC-EV, *n* = 5; IRI+ASC-EV, *n* = 3). Data represent mean ± SEM (* indicates statistical difference to SHAM group; # indicates statistical difference to IRI group; ** indicates statistical difference to IRI+iPSC-EV group; *p* <0.05 assessed by one-way ANOVA followed by Tukey’s test).

Oxidative stress is one of the main causes of kidney damage during IRI. Gene array analysis of kidney tissue revealed that iPSC-EV can modulate different genes associated with an oxidative stress response to IRI (Figure 6). We performed Gene Ontology (GO) enrichment analysis to determine the significant biological pathways associated with target genes modified by iPSC-EV treatment. As shown in Figure 6a, the group of genes modulated by iPSC-EV were mainly associated with molecular functions such as glutathione redox reactions, Nuclear factor erythroid 2-related factor 2 (NRF2)-mediated oxidative stress response, superoxide radical degradation, mitochondrial dysfunction, sirtuins pathways, and endothelin-1 signalling (Figure 6a), which are likely involved in the renal metabolic response during IRI [23].

In particular, the administration of iPSC-EV after ischemic insult resulted in an increase, in two iPSC-EV sources over three tested, of genes associated with the protection against the oxidative stress: glutathione S-transferase kappa 1 (GSTK1), selenoprotein P (SEPP1), superoxide dismutase 1 (SOD1), SOD3, thioredoxin 1 (TXN1), and thioredoxin reductase 2 (TXNRD2) (Figure 6b). All these genes were reduced in the IRI group with respect to the SHAM group, showing the impairment of the capacity of renal tissue to process the reactive oxygen species (ROS) during the occurrence of the ischemic damage (Table 1). Other genes such as iNOS, Nicotinamide-adenine dinucleotide phosphate (NADPH) oxidase organizer 1 (NOXO1), lactoperoxidase (LPO), and the dual oxidase 1 (DUOX1), associated with the formation of reactive oxygen species, resulted as being negatively regulated by iPSC-EV treatment with respect to IRI (Table 1). In order to compare the capacity of iPSC-EV and ASC-EV to regulate the genes associated with oxidative stress protection, we performed qRT-PCR analysis of selected genes. Obtained data showed that IRI strongly reduced SOD1 levels with respect to SHAM. Although both iPSC-EV and ASC-EV showed the capability to induce SOD1 expression, only the ASC-EV treatment reached significance (iPSC-EV, RQ = 1.5 and ASC-EV = 2.1 with respect to the IRI group) (Figure 6c). We also tested the expression of aldehyde oxidase-1 (AOX1), a Nrf2-dependent activated gene [24], that was significantly upregulated by both treatments with respect to IRI rats (iPSC-EV, ≈19-fold increase and ASC-EV, ≈22-fold increase) (Figure 6d). Because sirtuins are known to play major roles in protection against cellular stress and in controlling metabolic pathways [25], we evaluated the expression of some members of the sirtuins family that are associated with biological pathways overrepresented in the iPSC-EV-treated animals (Figure 6a,b). qRT-PCR analysis showed the capability of only iPSC-EV to significantly up-regulate sirtuin 1 (SIRT1) (iPSC-EV, ≈2-fold increase and ASC-EV, ≈1.5-fold increase, with respect to the IRI group) and sirtuin 2 (SIRT2) (iPSC-EV and ASC-EV, ≈1.5-fold increase, with respect to the IRI group) (Figure 6e,f, respectively). It is important to mention that IRI promoted the downregulation of several genes associated with protection against ROS, such as catalase (CAT), TXNRD2, peroxiredoxin 5 (PRDX5), SOD1, and SOD2 (Table S3).

## 4. Discussion

EV derived from stem cells have been shown to support tissue recovery, and investigations on new sources of EV could bring therapeutic alternatives in the treatment of kidney diseases. The results obtained in this work show, for the first time, that iPSC can be an interesting source of EV for treatment of AKI, capable of reducing renal cell death, tissue damage, and macrophage infiltration, and of ameliorating renal function. Part of iPSC-EV’s effects is associated with the protection of mitochondria from IRI damage, maintaining a functional mitochondrial mass despite the injury. Comparing the effects with ASC-EV, iPSC-EV presented similar beneficial properties and an even better outcome in the reduction of cell death. Gene array analysis revealed that iPSC-EV mechanism of action was associated with the regulation of key genes known to prevent damage caused by oxidative stress inside renal cells.

The therapeutic potential of MSC, along with their EV, has been the focus of several studies due to their beneficial effects in the AKI model [26,27,28]. However, the restricted availability of MSC, as in the cases of those derived from bone marrow, or the limited doubling properties, are limiting elements in the translation to clinical applications. To overcome such limitations, different groups have used MSC iPSC-derived as an alternative strategy for cellular therapy and as a source of EV in the treatment of multiple diseases, including AKI [29,30,31]. Despite iPSC having been shown also to attenuate AKI, no data have been presented on the therapeutic potential of EV secreted by these cells in kidney injury. In our experimental protocol, we decided to cultivate iPSC in serum-free composition-defined medium to guarantee the purity of the obtained EV and also to work in accordance with a perspective of clinical application where the use of animal serum would represent a limitation in the use of EV for the treatment. In our hands, iPSC secreted a heterogeneous population of EV, containing vesicles of different sizes that corresponded to exosomes and microvesicle populations. Moreover, a deep analysis in EV surface markers revealed that iPSC-EV carry several molecules that are associated with the pluripotent state of iPSC, confirming that EV derived from iPSC compartmentalize molecules from their cell of origin and are also of interest to some of their properties. Remarkably, among the molecules highly present in iPSC-EV, CD326—epithelial cell adhesion molecule (EpCAM)—has been reported to be upregulated in renal epithelial cells after IRI and to play an important role in renal regeneration [32]. Furthermore, CD133 (prominin-1) that is also carried by iPSC-EV has been described as being expressed in renal resident cells after damage. The regulatory role of CD133 seems to be mediated by Wnt/β-catenin signalling, inducing proliferation and limiting cell senescence [33]. Such compartmentalized molecules can confer specificity in the beneficial actions mediated by iPSC-EV, regulating key processes in renal recovery.

In order to evaluate iPSC-EV effects in the kidney, we performed a kidney subcapsular injection to optimize the EV delivery, as once only a fraction of intravenously administrated EV reached the kidney [34]. The administration of iPSC-EV just after the release of the vessel’s obstruction showed that it can act in the prevention of tissue damage progression in the critical phase of the reperfusion, instead of the recovery stage. In fact, the presence of EV administrated under the renal capsule could be mainly detected in the first 24 h after injection, supporting the early actions of EV [15]. As a result of iPSC-EV administration, the mitochondrial mass of renal cells was maintained, and more importantly, also the ΔΨm. The ΔΨm is an important element of energy mitochondrial storage that is used to generate ATP, and prolonged perturbation of its stability can result in loss of cell viability and trigger the pathological processes associated with oxidative stress [35]. The reduction of ATP intracellular levels provoked by chemical anoxia was partially reversed by the presence of ASC-EV, resulting in cell death protection [18]. Moreover, iPSC-EV showed a more efficient result in renal cell protection when compared to ASC-EV, although both EV presented similar responses in the maintenance of mitochondria mass and the ΔΨm. This data indicates that other elements, besides mitochondria protection, can be involved in renal cell death reduction promoted by iPSC-EV.

The role of iPSC-EV in renal tissue has also been associated with the modulation of the immune response. During AKI, circulating monocytes can be recruited to the kidney by secreted chemokines derived from the damaged tissue and differentiate into proinflammatory macrophages. The macrophages can increase reactive oxygen species generation and the apoptotic death rate of renal tubular cells [36]. Similarly, systemic depletion monocytes and macrophages prior to IRI attenuate morphologic kidney damage and partially re-establish the BUN and creatinine levels in the blood [37,38]. A similar response was obtained by the administration of iPSC-EV, resulting in the reduction of macrophage infiltration and the protection of renal function characterized by the reduced levels of BUN and creatinine in the blood. Interestingly, together with the reduction of macrophage infiltration, iPSC-EV administration led to an increase in the CD206 expression in renal tissue. CD206 is a marker of M2 macrophages, and such polarization has been described as occurring during the resolution phase of AKI, characterized by immunosuppression and tissue regeneration [39]. In this manner, iPSC-EV are capable of modulating the inflammatory environment and therefore promoting, also through this mechanism, the reduction of renal tissue damage.

Because mitochondria are mainly responsible for the intracellular ROS production during the reperfusion phase of IRI [40], and because the presence of iPSC-EV was shown to promote the protection of mitochondria, further investigation revealed that the mechanism associated with the beneficial effect of iPSC-EV is extended to the regulation of oxidative stress. Administration of iPSC-EV resulted in the modulation of several genes in the renal tissue associated with the protection against oxidative stress (SOD1, SOD3, SEPP1, TXN1, TXNRD2, GSTK1), and IPA analysis revealed that iPSC-EV regulated different cellular processes such as NRF2-mediated responses, superoxide radical degradation, mitochondrial dysfunction, production of nitric oxide, and reactive oxygen species in macrophages. The SODs are a group of enzymes responsible for catalyzing the conversion of superoxide into oxygen and hydrogen peroxide, and iPSC-EV promotes the increase of mRNA levels of *SOD1* and *SOD3.* The enzyme Sod1 is an important scavenger protein localized in the cytosol that acts against the oxidative stress by catalyzing the conversion of superoxide anion to hydrogen peroxide (H_2_O_2_). Posteriorly, the H_2_O_2_ can be reduced to water by other enzymes such as glutathione peroxidase or catalase [41]. Although the precise role of Sod3 requires further investigation, it is the most widely expressed isoform in the kidney and, therefore, it has an important role in the protection against oxidative stress during IRI [42]. In addition, Sod3 was reported to be a regulator of vascular function and is associated with the maintenance of renal blood flow after IRI [43].

The thioredoxin system seems to participate in the anti-ROS action modulated by iPSC-EV. Such a system is composed of ubiquitous thiol oxidoreductases that regulate cellular redox status [44]. In our model, iPSC-EV were shown to upregulate the expression of TXN1 and TXNRD2. Txn1 is a cytoplasmic enzyme and has been described to protect the kidney from oxidative stress, and overexpression of Txn1 resulted in the attenuation of the damage by IRI [44,45]. Txnrd2 is known to be an H_2_O_2_ scavenger and is responsible for maintaining the redox homeostasis by eliminating H_2_O_2_ and preserving mitochondria integrity [46]. In addition, Gstk1 is also another anti-ROS enzyme-modulated gene by iPSC-EV administration that is present in the mitochondria and possesses glutathione-conjugating activity towards halogenated aromatics [47,48] and, therefore, contributes to the reduction of the renal damage given by IRI. Another pro-regenerative molecule induced by iPSC-EV treatment is Sepp1, an extracellular glycoprotein that presents a dual role as a selenium supplier for the renal tissue and as an antioxidant, regulating late ROS accumulation [49]. Moreover, iPSC-EV treatment was also found to be able to induce the expression of SIRT1 and SIRT2, two members of the Sir2 (silent information regulator 2) family, a group of class III deacetylases. Sirtuins, whose pathway was overrepresented by the iPSC-EV treatment, are involved in cellular energy metabolism and control of the redox state [25]. In the kidneys, SIRT1 has been described as inhibiting renal cell apoptosis, inflammation, and fibrosis, and may regulate lipid metabolism, autophagy, blood pressure, and sodium balance [50]. iPSC-EV treatment can reduce some oxidative stress sensors such as iNOS, NOXO1, LPO, and DUOX1. Interestingly, it has been previously reported that post-AKI rats had an increased expression of other pro-oxidant genes such as LPO, myeloperoxidase, and DUOX1 [51]. Moreover, gene set enrichment analyses in IRI condition revealed that high DUOX1 expression is significantly correlated with the enrichment of immune pathways related to interferon (IFN)-alpha, IFN-gamma, and natural killer (NK) cell signalling [52].

Taken the data together, IPSC-EV were shown to be capable of acting on critical points of AKI, in terms of oxidative stress, inflammation, and cell death. Such properties show a new relevant application of iPSC in the treatment of kidney diseases, bypassing the risks associated with the use of the cell itself. In addition, iPSC can be explored as a new source of EV due to its properties associated with long-term survival, homogenous cell population, and high and fast expansion, that are known to be limited in MSC and critical under a therapeutic perspective [53]. Further studies investigating the entire composition of iPSC-EV would be valuable in better understanding their safety and potential in tissue regeneration as a therapeutic tool.

## 5. Conclusions

This work shows that iPSC-EV have a protective role in kidney IRI, acting in different aspects associated with tissue damage. Gene array analysis revealed that the main mechanisms associated with the beneficial effects of iPSC-EV is based on the protection of mitochondria, as well as reduction of oxidative stress and inflammation. Administration of iPSC-EV presented as similar or in some aspects higher efficiency in the kidney protection when compared to ASC-EV. Therefore, iPSC can be considered as an alternative source of EV with therapeutic properties to be explored for the treatment of kidney diseases.

## Figures and Tables

**Table 1:** Genes modulated by iPSC-EV treatment during IRI and associated with oxidative stress in renal tissue (iPSC-EV with respect to IRI).

Fold Change with respect to IRI	Symbol	Gene Name	Location
13.0	RAG2	recombination activating 2	Nucleus
8.6	NOXA1	NADPH oxidase activator 1	Plasma Membrane
3.5	UCP3	uncoupling protein 3	Cytoplasm
2.7	NGB	neuroglobin	Cytoplasm
2.1	Mpo	myeloperoxidase	Extracellular Space
1.8	Hba-a2/Hba1	hemoglobin, alpha 1	Cytoplasm
1.8	AOX1	aldehyde oxidase 1	Cytoplasm
1.8	DUOX2	dual oxidase 2	Plasma Membrane
1.7	GPX6	glutathione peroxidase 6	Extracellular Space
1.7	TPO	thyroid peroxidase	Plasma Membrane
1.7	SOD1	superoxide dismutase 1	Cytoplasm
1.6	NOX4	NADPH oxidase 4	Cytoplasm
1.6	ERCC6	ERCC excision repair 6, chromatin remodeling factor	Nucleus
1.6	PTGS1	prostaglandin-endoperoxide synthase 1	Cytoplasm
1.6	CYBA	cytochrome b-245 alpha chain	Cytoplasm
1.6	CCL5	C-C motif chemokine ligand 5	Extracellular Space
1.6	EHD2	EH domain containing 2	Nucleus
1.6	PSMB5	proteasome subunit beta 5	Cytoplasm
1.6	TXNRD2	thioredoxin reductase 2	Cytoplasm
1.5	SLC38A1	solute carrier family 38 member 1	Plasma Membrane
1.5	SOD3	superoxide dismutase 3	Extracellular Space
1.5	EPX	eosinophil peroxidase	Cytoplasm
1.5	PARK7	Parkinsonism associated deglycase	Nucleus
1.5	TXN	thioredoxin	Cytoplasm
1.5	GSTK1	glutathione S-transferase kappa 1	Cytoplasm
−1.6	NOXO1	NADPH oxidase organizer 1	Plasma Membrane
−1.6	GSTP1	glutathione S-transferase pi 1	Cytoplasm
−1.8	GPX5	glutathione peroxidase 5	Extracellular Space
−1.9	ALB	albumin	Extracellular Space
−3.0	LPO	lactoperoxidase	Extracellular Space
−8.0	NOS2	nitric oxide synthase 2	Cytoplasm
−11.1	DUOX1	dual oxidase 1	Plasma Membrane
−11.4	MB	myoglobin	Cytoplasm
−21.5	HSPA1A/HSPA1B	heat shock protein family A (Hsp70) member 1A	Cytoplasm

## Supplementary Materials

The following are available online at https://www.mdpi.com/2073-4409/9/2/453/s1, Table S1: Raw data of oxidative stress gene arrays from renal tissue in the different experimental conditions in the in vivo model. Table S2:. List of the primers used in the qRT-PCR analysis. Table S3: Modulated genes associated with oxidative stress in renal tissue, modulated by IRI (SHAM with respect to IRI).

## References

[1] Peired A.J., Sisti A., Romagnani P. (2016). Mesenchymal Stem Cell-Based Therapy for Kidney Disease: A Review of Clinical Evidence. Stem Cells Int..

[2] Yasuda S., Kusakawa S., Kuroda T., Miura T., Tano K., Takada N., Matsuyama S., Matsuyama A., Nasu M., Umezawa A. (2018). Tumorigenicity-associated characteristics of human iPS cell lines. PLoS ONE..

[3] Tögel F., Zhang P., Hu Z., Westenfelder C. (2009). VEGF is a mediator of the renoprotective effects of multipotent marrow stromal cells in acute kidney injury. J. Cell Mol. Med..

[4] Colombo M., Raposo G., Théry C. (2014). Biogenesis, secretion, and intercellular interactions of exosomes and other extracellular vesicles. Annu. Rev. Cell Dev. Biol..

[5] Yáñez-Mó M., Siljander P.R., Andreu Z., Zavec A.B., Borràs F.E., Buzas E.I., Buzas K., Casal E., Cappello F., Carvalho J. (2015). Biological properties of extracellular vesicles and their physiological functions. J. Extracell Vesicles.

[6] Bruno S., Bussolati B., Grange C., Collino F., di Cantogno L.V., Herrera M.B., Biancone L., Tetta C., Segoloni G., Camussi G. (2009). Isolation and characterization of resident mesenchymal stem cells in human glomeruli. Stem Cells Dev..

[7] Lindoso R.S., Collino F., Bruno S., Araujo D.S., Sant’Anna J.F., Tetta C., Provero P., Quesenberry P.J., Vieyra A., Einicker-Lamas M. (2014). Extracellular vesicles released from mesenchymal stromal cells modulate miRNA in renal tubular cells and inhibit ATP depletion injury. Stem Cells Dev..

[8] Lindoso R.S., Lopes J.A., Binato R., Abdelhay E., Takiya C.M., Miranda K.R., Lara L.S., Viola A., Bussolati B., Vieyra A. (2019). Adipose mesenchymal cells-derived EVs alleviate DOCA-salt-induced hypertension by promoting cardio-renal protection. Mol. Ther. Methods Clin. Dev..

[9] Grange C., Tritta S., Tapparo M., Cedrino M., Tetta C., Camussi G., Brizzi M.F. (2019). Stem cell-derived extracellular vesicles inhibit and revert fibrosis progression in a mouse model of diabetic nephropathy. Sci. Rep..

[10] Takahashi K., Yamanaka S. (2006). Induction of pluripotent stem cells from mouse embryonic and adult fibroblast cultures by defined factors. Cell.

[11] Martin U. (2017). Therapeutic Application of Pluripotent Stem Cells: Challenges and Risks. Front. Med..

[12] Lee P.Y., Chien Y., Chiou G.Y., Lin C.H., Chiou C.H., Tarng D.C. (2012). Induced pluripotent stem cells without c-Myc attenuate acute kidney injury via downregulating the signaling of oxidative stress and inflammation in ischemia-reperfusion rats. Cell Transplant..

[13] Wang Y., Zhang L., Li Y., Chen L., Wang X., Guo W., Zhang X., Qin G., He S.H., Zimmerman A. (2015). Exosomes/microvesicles from induced pluripotent stem cells deliver cardioprotective miRNAs and prevent cardiomyocyte apoptosis in the ischemic myocardium. Int. J. Cardiol..

[14] Sochacki J., Devalle S., Reis M., Mattos P., Rehen S. (2016). Generation of urine iPS cell lines from patients with Attention Deficit Hyperactivity Disorder (ADHD) using a non-integrative method. Stem Cell Res..

[15] Takahashi K., Tanabe K., Ohnuki M., Narita M., Ichisaka T., Tomoda K., Yamanaka S. (2007). Induction of pluripotent stem cells from adult human fibroblasts by defined factors. Cell.

[16] Kasai-Brunswick T.H., Silva Dos Santos D., Ferreira R.P., Araujo D.S., Dias G.M., Coutinho J.L.A., Cruz F.E.S.F., Sternick E.B., Gubert F., Oliveira J.C.G. (2018). Generation of patient-specific induced pluripotent stem cell lines from one patient with Jervell and Lange-Nielsen syndrome, one with type 1 long QT syndrome and two healthy relatives. Stem Cell Res..

[17] Théry C., Witwer K.W., Aikawa E., Alcaraz M.J., Anderson J.D., Andriantsitohaina R., Antoniou A., Arab T., Archer F., Atkin-Smith G.K. (2018). Minimal information for studies of extracellular vesicles 2018 (MISEV2018): A position statement of the International Society for Extracellular Vesicles and update of the MISEV2014 guidelines. J Extracell. Vesicles.

[18] Collino F., Lopes J.A., Corrêa S., Abdelhay E., Takiya C.M., Wendt C.H.C., de Miranda K.R., Vieyra A., Lindoso R.S. (2019). Adipose-Derived Mesenchymal Stromal Cells Under Hypoxia: Changes in Extracellular Vesicles Secretion and Improvement of Renal Recovery after Ischemic Injury. Cell Physiol. Biochem..

[19] Bruno S., Pasquino C., Herrera Sanchez M.B., Tapparo M., Figliolini F., Grange C., Chiabotto G., Cedrino M., Deregibus M.C., Tetta C. (2020). HLSC-derived extracellular vesicles attenuate liver fibrosis and inflammation in a murine model of non-alcoholic steatohepatitis. Mol. Ther..

[20] Bruno S., Grange C., Deregibus M.C., Calogero R.A., Saviozzi S., Collino F., Morando L., Busca A., Falda M., Bussolati B. (2009). Mesenchymal stem cell-derived microvesicles protect against acute tubular injury. J. Am. Soc. Nephrol..

[21] Yamanaka S. (2009). A fresh look at iPS cells. Cell..

[22] Lemasters J.J., Nieminen A.L., Qian T., Trost L.C., Elmore S.P., Nishimura Y., Crowe R.A., Cascio W.E., Bradham C.A., Brenner D.A. (1998). The mitochondrial permeability transition in cell death: A common mechanism in necrosis, apoptosis and autophagy. Biochim. Biophys. Acta.

[23] Martin J.L., Gruszczyk A.V., Beach T.E., Murphy M.P., Saeb-Parsy K. (2019). Mitochondrial mechanisms and therapeutics in ischaemia reperfusion injury. Pediatr. Nephrol..

[24] Maeda K., Ohno T., Igarashi S., Yoshimura T., Yamashiro K., Sakai M. (2012). Aldehyde oxidase 1 gene is regulated by Nrf2 pathway. Gene.

[25] Poulose N., Raju R. (2015). Sirtuin regulation in aging and injury. Biochim. Biophys. Acta.

[26] Bruno S., Tapparo M., Collino F., Chiabotto G., Deregibus M.C., Soares Lindoso R., Neri F., Kholia S., Giunti S., Wen S. (2017). Renal Regenerative Potential of Different Extracellular Vesicle Populations Derived from Bone Marrow Mesenchymal Stromal Cells. Tissue Eng. Part A.

[27] Yagi H., Soto-Gutierrez A., Kitagawa Y., Tilles A.W., Tompkins R.G., Yarmush M.L. (2010). Bone marrow mesenchymal stromal cells attenuate organ injury induced by LPS and burn. Cell Transplant..

[28] Herrera M.B., Bussolati B., Bruno S., Morando L., Mauriello-Romanazzi G., Sanavio F., Stamenkovic I., Biancone L., Camussi G. (2007). Exogenous mesenchymal stem cells localize to the kidney by means of CD44 following acute tubular injury. Kidney Int..

[29] Luzzani C.D., Miriuka S.G. (2017). Pluripotent Stem Cells as a Robust Source of Mesenchymal Stem Cells. Stem Cell Rev. Rep..

[30] Yuan X., Li D., Chen X., Han C., Xu L., Huang T., Dong Z., Zhang M. (2017). Extracellular vesicles from human-induced pluripotent stem cell-derived mesenchymal stromal cells (hiPSC-MSCs) protect against renal ischemia/reperfusion injury via delivering specificity protein (SP1) and transcriptional activating of sphingosine kinase 1 and inhibiting necroptosis. Cell Death Dis..

[31] Sheyn D., Ben-David S., Shapiro G., De Mel S., Bez M., Ornelas L., Sahabian A., Sareen D., Da X., Pelled G. (2016). Human induced pluripotent stem cells differentiate into functional mesenchymal stem cells and repair bone defects. Stem Cells Transl. Med..

[32] Trzpis M., McLaughlin P.M., van Goor H., Brinker M.G., van Dam G.M., de Leij L.M., Popa E.R., Harmsen M.C. (2008). Expression of EpCAM is up-regulated during regeneration of renal epithelia. J. Pathol..

[33] Brossa A., Papadimitriou E., Collino F., Incarnato D., Oliviero S., Camussi G., Bussolati B. (2018). Role of CD133 molecule in Wnt response and renal repair. Stem Cells Transl. Med..

[34] Grange C., Tapparo M., Bruno S., Chatterjee D., Quesenberry P.J., Tetta C., Camussi G. (2014). Biodistribution of mesenchymal stem cell-derived extracellular vesicles in a model of acute kidney injury monitored by optical imaging. Int. J. Mol. Med..

[35] Izyumov D.S., Avetisyan A.V., Pletjushkina O.Y., Sakharov D.V., Wirtz K.W., Chernyak B.V., Skulachev V.P. (2004). “Wages of fear”: Transient threefold decrease in intracellular ATP level imposes apoptosis. Biochim. Biophys. Acta.

[36] Cao Q., Harris D.C., Wang Y. (2015). Macrophages in kidney injury, inflammation, and fibrosis. Physiology.

[37] Jo S.K., Sung S.A., Cho W.Y., Go K.J., Kim H.K. (2006). Macrophages contribute to the initiation of ischaemic acute renal failure in rats. Nephrol. Dial. Transplant..

[38] Day Y.J., Huang L., Ye H., Linden J., Okusa M.D. (2005). Renal ischemia-reperfusion injury and adenosine 2A receptor-mediated tissue protection: Role of macrophages. Am. J. Physiol. Renal Physiol..

[39] Chen T., Cao Q., Wang Y., Harris D.C.H. (2019). M2 macrophages in kidney disease: Biology, therapies, and perspectives. Kidney Int..

[40] Granger D.N., Kvietys P.R. (2015). Reperfusion injury and reactive oxygen species: The evolution of a concept. Redox Biol..

[41] Fridovich I. (1995). Superoxide radical and superoxide dismutases. Annu. Rev. Biochem..

[42] Schneider M.P., Sullivan J.C., Wach P.F., Boesen E.I., Yamamoto T., Fukai T., Harrison D.G., Pollock D.M., Pollock J.S. (2010). Protective role of extracellular superoxide dismutase in renal ischemia/reperfusion injury. Kidney Int..

[43] Strålin P., Karlsson K., Johansson B.O., Marklund S.L. (1995). The interstitium of the human arterial wall contains very large amounts of extracellular superoxide dismutase. Arterioscler. Thromb. Vasc. Biol..

[44] Kasuno K., Nakamura H., Ono T., Muso E., Yodoi J. (2003). Protective roles of thioredoxin, a redox-regulating protein, in renal ischemia/reperfusion injury. Kidney Int..

[45] Kim Y.C., Masutani H., Yamaguchi Y., Itoh K., Yamamoto M., Yodoi J. (2001). Hemin-induced activation of the thioredoxin gene by Nrf2. A differential regulation of the antioxidant responsive element by a switch of its binding factors. J. Biol. Chem..

[46] Kiermayer C., Northrup E., Schrewe A., Walch A., de Angelis M.H., Schoensiegel F., Zischka H., Prehn C., Adamski J., Bekeredjian R. (2015). Heart-Specific Knockout of the Mitochondrial Thioredoxin Reductase (Txnrd2) Induces metabolic and contractile dysfunction in the aging myocardium. J. Am. Heart Assoc..

[47] Marí M., Morales A., Colell A., García-Ruiz C., Fernández-Checa J.C. (2009). Mitochondrial glutathione, a key survival antioxidant. Antioxid. Redox Signal..

[48] Morel F., Aninat C. (2011). The glutathione transferase kappa family. Drug Metab. Rev..

[49] Burk R.F., Hill K.E. (2009). Selenoprotein P-expression, functions, and roles in mammals. Biochim. Biophys. Acta.

[50] Kitada M., Kume S., Takeda-Watanabe A., Kanasaki K., Koya D. (2013). Sirtuins and renal diseases: Relationship with aging and diabetic nephropathy. Clin. Sci..

[51] Basile D.P., Leonard E.C., Beal A.G., Schleuter D., Friedrich J. (2012). Persistent oxidative stress following renal ischemia-reperfusion injury increases ANG II hemodynamic and fibrotic activity. Am. J. Physiol. Renal Physiol..

[52] Cho S.Y., Kim S., Son M.J., Kim G., Singh P., Kim H.N., Choi H.G., Yoo H.J., Ko Y.B., Lee B.S. (2019). Dual oxidase 1 and NADPH oxidase 2 exert favorable effects in cervical cancer patients by activating immune response. BMC Cancer.

[53] Lavon N., Zimerman M., Itskovitz-Eldor J. (2018). Scalable expansion of pluripotent stem cells. Adv. Biochem. Eng. Biotechnol..

