# Supplementary material for: Extracellular Vesicles Derived from Induced Pluripotent Stem Cells Promote Renoprotection in Acute Kidney Injury Model
Source: Cells. 2020 Feb 17;9(2):453. doi: 10.3390/cells9020453 (PMC7072760; doi:10.3390/cells9020453)
Supplement: Supplementary file 1 [file cells-09-00453-s001.zip › Supplementary files/Table S2.docx]

| Genes | Forward | Reverse |
| --- | --- | --- |
| hSOX2 | TGCGAGCGCTGCACAT | GCAGCGTGTACTTATCCTTCTTCA |
| hOCT4 | GAAAGCGAACCAGTATCGAAAC | ACACTCGGACCACATCTTCTC |
| hC-MYC | CAGCGACTCTGAGGGGAACA | TGAGGAGGTTTGCTGTGGC |
| hNANOG | ACAACTGGCCGAAGAATAGCA | GGTTCCCAGTCGGGTTCAC |
| hACTIN | TGAAGATCAAGATCATTGCTCCTC | CACATCTGCTGGAAGGTGGAC |
| riNOS | CTCAGGCTTGGGTCTTGTTAG | TGTTGTTGGGCTGGGAATAG |
| rCD206 | GACGGACGAGGAGTTCATTATAC | GTTGGAGAGATAGGCACAGAAG |
| rSOD1 | AAGAGAGGCATGTTGGAGACC | ACGGCCAATGATGGAATGCT |
| rAOX1 | TGCTGGAAGGTGGCATTGA | CTCATCCACCGCAATTTGGT |
| rSIRT1 | GAAACCCTCAATTTCTGTTCTGCT | AATGCGATGCTGACTTCCTTCT |
| rSIRT2 | TCCGCAGGAATCCCTGACT | GTATGGAAGGTGGTATTTCTCCAAGT |
| rACTIN | ACCGTGAAAAGATGACCCAGAT | CACAGCCTGGATGGCTACGT |
