# Supplementary material for: Extracellular Vesicles Derived from Induced Pluripotent Stem Cells Promote Renoprotection in Acute Kidney Injury Model
Source: Cells. 2020 Feb 17;9(2):453. doi: 10.3390/cells9020453 (PMC7072760; doi:10.3390/cells9020453)
Supplement: Supplementary file 1 [file cells-09-00453-s001.zip › Supplementary files/Table S3.docx]

| Fold Change in respect to IRI | Symbol | Gene Name | Location |
| --- | --- | --- | --- |
| 16.5 | ALB | albumin | Extracellular Space |
| 9.7 | NOXA1 | NADPH oxidase activator 1 | Plasma Membrane |
| 9.0 | GCLC | glutamate-cysteine ligase catalytic subunit | Cytoplasm |
| 7.5 | Hba-a2/Hba1 | hemoglobin. alpha 1 | Cytoplasm |
| 6.4 | NOX4 | NADPH oxidase 4 | Cytoplasm |
| 5.7 | KRT1 | keratin 1 | Cytoplasm |
| 4.8 | GPX3 | glutathione peroxidase 3 | Extracellular Space |
| 4.6 | CAT | catalase | Cytoplasm |
| 3.3 | PRDX5 | peroxiredoxin 5 | Cytoplasm |
| 3.1 | CCL5 | C-C motif chemokine ligand 5 | Extracellular Space |
| 3.0 | NGB | neuroglobin | Cytoplasm |
| 2.8 | APOE | apolipoprotein E | Extracellular Space |
| 2.8 | SOD1 | superoxide dismutase 1 | Cytoplasm |
| 2.8 | GSTK1 | glutathione S-transferase kappa 1 | Cytoplasm |
| 2.8 | IFT172 | intraflagellar transport 172 | Extracellular Space |
| 2.7 | PTGS2 | prostaglandin-endoperoxide synthase 2 | Cytoplasm |
| 2.6 | CCS | copper chaperone for superoxide dismutase | Cytoplasm |
| 2.5 | FMO2 | flavin containing monooxygenase 2 | Cytoplasm |
| 2.4 | PARK7 | Parkinsonism associated deglycase | Nucleus |
| 2.4 | PRDX3 | peroxiredoxin 3 | Cytoplasm |
| 2.3 | Mpo | myeloperoxidase | Extracellular Space |
| 2.3 | TXNRD2 | thioredoxin reductase 2 | Cytoplasm |
| 2.3 | IDH1 | isocitrate dehydrogenase (NADP(+)) 1. cytosolic | Cytoplasm |
| 2.2 | CYBA | cytochrome b-245 alpha chain | Cytoplasm |
| 2.2 | SOD2 | superoxide dismutase 2 | Cytoplasm |
| 2.1 | ALS2 | alsin Rho guanine nucleotide exchange factor ALS2 | Cytoplasm |
| 2.1 | ERCC6 | ERCC excision repair 6. chromatin remodeling factor | Nucleus |
| 2.1 | APC | APC regulator of WNT signaling pathway | Nucleus |
| 2.0 | FTH1 | ferritin heavy chain 1 | Cytoplasm |
| 1.9 | EPX | eosinophil peroxidase | Cytoplasm |
| 1.9 | GSR | glutathione-disulfide reductase | Cytoplasm |
| 1.8 | PSMB5 | proteasome subunit beta 5 | Cytoplasm |
| 1.7 | SLC38A1 | solute carrier family 38 member 1 | Plasma Membrane |
| 1.7 | PRDX1 | peroxiredoxin 1 | Cytoplasm |
| 1.6 | TXNIP | thioredoxin interacting protein | Cytoplasm |
| 1.5 | FANCC | FA complementation group C | Nucleus |
| 1.5 | PTGS1 | prostaglandin-endoperoxide synthase 1 | Cytoplasm |
| 1.5 | PRDX6 | peroxiredoxin 6 | Cytoplasm |
| 1.5 | SOD3 | superoxide dismutase 3 | Extracellular Space |
| 1.5 | PRDX2 | peroxiredoxin 2 | Cytoplasm |
| 1.5 | GPX4 | glutathione peroxidase 4 | Cytoplasm |
| -1.5 | LPO | lactoperoxidase | Extracellular Space |
| -1.7 | CYGB | cytoglobin | Cytoplasm |
| -1.9 | NCF1 | neutrophil cytosolic factor 1 | Cytoplasm |
| -2.2 | UCP2 | uncoupling protein 2 | Cytoplasm |
| -2.3 | GPX7 | glutathione peroxidase 7 | Cytoplasm |
| -3.1 | GSTP1 | glutathione S-transferase pi 1 | Cytoplasm |
| -3.1 | NCF2 | neutrophil cytosolic factor 2 | Cytoplasm |
| -3.7 | GPX2 | glutathione peroxidase 2 | Cytoplasm |
| -4.0 | SRXN1 | sulfiredoxin 1 | Cytoplasm |
| -5.5 | HMOX1 | heme oxygenase 1 | Cytoplasm |
| -6.2 | VIM | vimentin | Cytoplasm |
| -9.0 | NOXO1 | NADPH oxidase organizer 1 | Plasma Membrane |
| -9.9 | DUOX1 | dual oxidase 1 | Plasma Membrane |
| -16.5 | MB | myoglobin | Cytoplasm |
| -16.8 | UCP3 | uncoupling protein 3 | Cytoplasm |
| -64.0 | NOS2 | nitric oxide synthase 2 | Cytoplasm |
